# Mechanical properties of the gastrocnemius muscle‐tendon unit in male athletic high jumpers

**DOI:** 10.14814/phy2.70370

**Published:** 2025-05-09

**Authors:** Toshihide Fujimori, Natsuki Sado

**Affiliations:** ^1^ Graduate School of Comprehensive Human Sciences University of Tsukuba Tsukuba Japan; ^2^ Japan Society for the Promotion of Science Tokyo Japan; ^3^ Institute of Health and Sport Sciences University of Tsukuba Tsukuba Japan; ^4^ Advanced Research Initiative for Human High Performance University of Tsukuba Tsukuba Japan

**Keywords:** biomechanics, in vivo, muscle tendon unit, performance, skeletal muscle, tendon stiffness

## Abstract

Tendon compliance facilitates power exertion during stretch‐shortening cycle exercises through muscle‐tendon interaction. Tendons stiffen in response to mechanical loadings, and their stiffness sometimes affects motor performance, but no consensus has been reached yet. We investigated the gastrocnemius muscle‐tendon properties of 10 male amateur high jumpers and 14 untrained males. Participants performed maximum voluntary contraction (MVC) of ankle plantar flexion. We measured the maximum joint torque and Achilles tendon stiffness using a torque dynamometer for force measurement, an ultrasound apparatus to track tendon elongation, and a motion capture system to correct joint rotation. High jumpers exerted significantly greater MVC torque than untrained individuals (152.8 ± 31.8 vs. 103.6 ± 18.9 Nm). Tendon stiffness did not significantly differ between groups (287.3 ± 90.9 vs. 258.4 ± 85.6 N/mm). This suggests that strengthening muscles and stiffening tendons may independently adapt through high jump training. In high jumpers, high jump personal best record significantly correlated with MVC torque (*r* = 0.73) but not significantly correlated with tendon stiffness (*r* = −0.07). Muscle force exertion ability enhanced by training should be important for improving high jump performance, while tendon stiffening is not necessary for performance. We suggest that humans may inherently have adequate tendon properties for jumping, even without specific training.

## 1 INTRODUCTION

Tendon is viscoelastic tissue, which connects muscle and bone in series and transmits muscle force to the bone. The largest tendon in humans is the Achilles tendon (AT), a component of the ankle plantar flexors muscle‐tendon unit (MTU). The ankle plantar flexors are the main power generators during the stance phase in locomotion (Debaere et al., 2015). In locomotion, the MTU shows stretch‐shortening cycle (SSC) action (Komi, 1984), in which the compliant AT stores and releases elastic strain energy. The AT takes most of the whole MTU length change (Fukashiro et al., 2006; Fukunaga et al., 2001), leading to slow and minimum length changes in the muscle. Muscles can exert large force at optimum length and slow‐shortening conditions (Hill, 1938). Due to such force‐length‐velocity relationships in muscles, tendon compliance facilitates muscle force exertion (Bohm et al., 2021; Fukashiro et al., 2006). Thus, tendon compliance can affect human motor performance via muscle‐tendon interaction, whereas the relationship between MTU properties and motor performance remains controversial (Kubo et al., 2015; Passini et al., 2021; Pentidis et al., 2020).

MTU has an adaptability to mechanical loading (Bohm et al., 2015). High‐intensity resistance training induces a much greater increase in tendon stiffness than the increase in isometric maximum voluntary contraction (MVC) torque (Albracht & Arampatzis, 2013; Kubo et al., 2010). This implies a decrease in relative tendon compliance to muscle strength. Meanwhile, the MTU adaptation to plyometric training is controversial; some plyometric training interventions increased tendon stiffness (Fouré et al., 2010; Wu et al., 2010), while others did not (Houghton et al., 2013; Kubo et al., 2017). These controversial results may be caused by differences in the loading magnitude and in the intervention duration (Bohm et al., 2015), but plyometric training may stiffen tendon less than resistance training. Training interventions have rarely been reported to increase tendon compliance (i.e., decreases tendon stiffness). Tendon seems to stiffen in response to the loadings. However, it must be considered that these intervention studies are limited to short‐term (<6 months). A systematic review (Wiesinger et al., 2015) noted the lack of reports on tendon adaptation for longer than 14 weeks. Considering the poor tissue turnover in tendon (Heinemeier et al., 2013), long‐term adaptation could be different from short‐term adaptation.

Long‐term muscle and tendon adaptation to the mechanical loadings has been indirectly examined by comparing athletes with untrained populations [see review (Bohm et al., 2015)], although the MTU properties in athletes could be affected by intrinsic factors. Representative motor tasks requiring the ankle plantar flexion torque and power exertions are sprinting (Schache et al., 2011) and jumping (Sado et al., 2018). Sprinters have been reported to exert greater MVC ankle plantar flexion torque and to have stiffer AT than the untrained population (e.g., Arampatzis et al., 2007; Epro et al., 2021). To the best of our knowledge, only Karamanidis and Epro (2020) showed athletic jumpers (including 17 long jumpers, 22 high jumpers, 11 triple jumpers, and 17 pole vaulters) with greater torque and stiffer AT than the untrained population; however, it is debatable whether all jumping events lead to similar MTU adaptation. Based on that the AT takes most of the whole ankle plantar flexors MTU length change during SSC (Fukashiro et al., 2006; Fukunaga et al., 2001), the optimal AT properties might depend on the temporal characteristics of the motor task. The contact time in athletic high jump [≂0.2 s (Isolehto et al., 2007)] is approximately twice that in sprinting [≤0.1 s (Bezodis et al., 2008)] and in long jump [≂0.12 s (Wilson et al., 2011)], and even in world‐elite high jumpers, the contact time differs by more than 20% (Isolehto et al., 2007). Kuitunen et al. (2002) showed a strong correlation between ankle rotational stiffness and contact time in sprinting and suggested that the AT stiffness may influence the ground contact time. High jumpers vertically push the ground as forcefully for a relatively long time in order to jump higher, whereas sprinters and jumpers in horizontal jumping events need to get their foot off the ground quickly. Thus, high jumping requires great torque but may not require stiff AT. However, no studies have examined MTU properties in high jumpers and their relevance to performance. If humans can strengthen the ankle plantar flexors without stiffening the AT, high jumpers may acquire a large torque exertion ability without stiffening in the AT.

We aimed to compare the MTU properties in male high jumpers and untrained males to investigate MTU adaptation, and to clarify the suitable MTU properties for the high jump. We hypothesized that in the high jump, a large MVC torque is required to generate large torque during take‐off, but the tendon does not need to be stiff. If the hypothesis is proven, it will be shown that humans can strengthen their muscles while maintaining tendon compliance.

## 2 METHODS

### 2.1 Participants

According to an a priori power analysis for an independent two‐tailed *t*‐test (type 1 error (α‐level) of 0.05 and power (1−β) of 0.8) and Cohen's *d* = 1.2: very large (Sawilowsky, 2009), the total sample size was estimated to be at least 24. The participants were 24 young men, including 14 untrained male individuals [controls, age (mean ± standard deviation): 23.6 ± 2.1 years] and 10 amateur male high jump athletes (high jumpers, age: 20.1 ± 1.6 years). The athletic career, training volume, and personal best record were 7.7 ± 2.4 years [range: 4–12 years], 12.8 ± 2.7 h/week [range: 8–17 h/week], and 2.03 ± 0.11 m [range: 1.84–2.16 m], respectively. The criterion for the control group was that they had not participated in any organized program of regular exercise with training of the lower extremities for at least 2 years prior to the test. For recruiting the controls, it is ideal to match both body height and mass; however, it is impractical to match the body height of untrained individuals to high jumpers who are extremely tall individuals. We recruited controls with a body mass similar to that of the high jumpers. The a priori exclusion criterion was set such that the participants had prior AT injuries (e.g., ruptures and tendinopathy) during the preceding 6 months, as the presence of such injuries may affect the findings; however, no participant was excluded. All participants signed an informed consent form to participate in the study prior to the experiment. The experimental procedure was approved by the Human Subjects Committee of University of Tsukuba, Japan (reference number: 021‐95).

### 2.2 Experimental procedure

All measurements were performed on the nondominant leg (the opposite leg used to kick the football), as high jumpers take off using their nondominant leg. We examined the mechanical properties of the medial gastrocnemius (MG)–AT unit by combining measurements of ankle joint torque and tendon elongation during ramp trials.

We measured body height and mass to the nearest 1 mm and 50 g using a height scale (MZ10042, ADE Germany, Hamburg, Germany) and a weight scale (HD‐664, TANITA, Tokyo, Japan), respectively. We measured the shank length as the distance from the articular cleft between the femur and tibia to the lateral malleolus using a tape measure in the standing position. To measure the resting AT length and cross‐sectional area (CSA), the participants lay prone (Mogi et al., 2018) on top of the dynamometer (Biodex System 4, Biodex Medical Systems, Inc., New York, USA). The resting AT length and cross‐sectional area (CSA) were measured with the hip, knee, and ankle in an anatomical position (Mogi et al., 2018) using an ultrasound apparatus (MyLab25, Esaote, Genoa, Italy; equipped with LA523 linear array probe, 4–13 MHz, 46 mm). The resting AT length was defined as the distance from the calcaneal tuberosities to the MG–AT junction, with both locations identified using B‐mode ultrasound longitudinal images. To measure the AT CSA, three B‐mode ultrasound transverse images were obtained at the lateral malleolus height (Kubo et al., 2020) using the ultrasound apparatus.

Participants were prone on the dynamometer in the same position as for the CSA measurement. Their body was secured using a strap belt. Retroreflective markers were attached to the fifth metatarsal, lateral malleolus, and fibula apex. The axis of ankle plantar flexion was carefully aligned with the axis of rotation of the dynamometer, and the ankle joint was secured tightly to the footplate connected to its arm using a ratchet tie‐down strap. The ultrasound probe was attached to the skin at 30% of the shank length using a custom‐made polystyrene‐foam attachment with adhesive tape. An echo‐absorptive marker was placed between the skin and the probe to check whether the probe did not slide during trials.

Prior to the ramp trials, all participants were subjected to 3–5 s plantar flexion contraction trials of 30%, 50%, 70%, 90%, and 100% MVC for preconditioning of the tendon (Maganaris, 2003). After the preconditioning trials, the participants performed ramp trials in which the ankle torque was gradually increased from the relaxed state to the MVC within 5 s (Kubo et al., 2012). The participants were provided with real‐time torque feedback from the monitor. The ramp trials were repeated twice, with at least 3‐min intervals between trials, to prevent fatigue. If the two MVC torques differed by >10%, the participant performed a third trial. After the ramp trials, the participants performed a passive trial in which the ankle joint was rotated fifth at 5°/s from 10° dorsiflexion to 20° plantar flexion (Kubo et al., 2010).

The ankle torque was measured using a torque dynamometer. The raw torque signal from the dynamometer was recorded at 2000 Hz using an analogue–digital converter (cDAQ‐9178, National Instruments, Austin, USA). A 3‐camera motion capture system (Mac 3D System, Motion Analysis Corporation, Santa Rosa, USA) recorded the three‐dimensional position coordinates of the reflective markers at 200 Hz. An ultrasound apparatus (HI VISION Preirus, Hitachi Aloka Medical, Ltd., Tokyo, Japan; equipped with EUP‐L53, linear array probe, 7.5–13.0 MHz, 64 mm) was used to obtain longitudinal ultrasound images of the MG. The ultrasound images were recorded on the apparatus's internal storage at 67 Hz. We input the signal from a custom‐made synchronization device into the electrocardiogram signal recording function in the ultrasound apparatus and the analogue–digital converter to synchronize the torque signal, ultrasound images, and marker coordinate data for subsequent analysis.

### 2.3 Data processing

**FIGURE 1:**
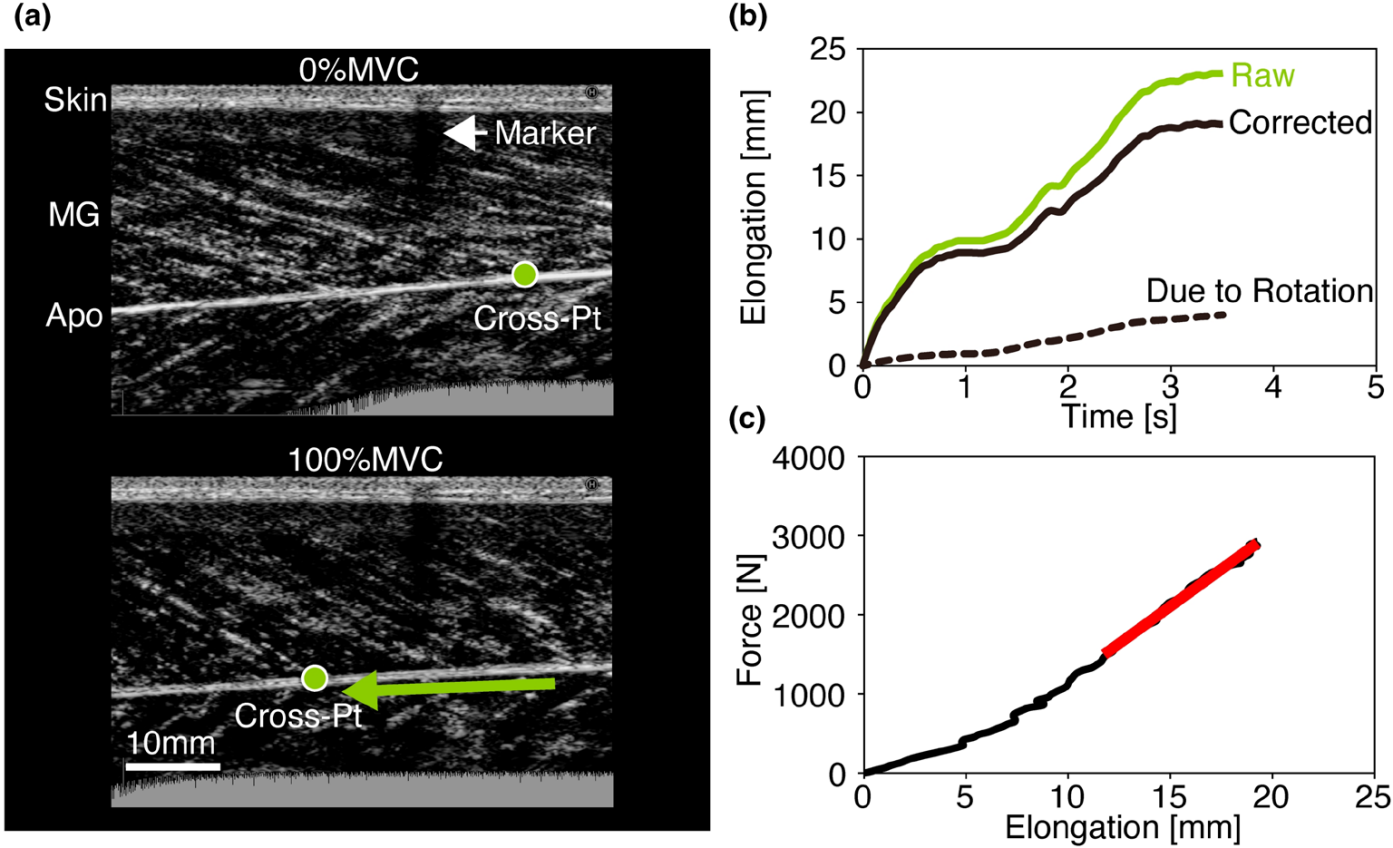
Data processing. (a) An example of ultrasound images of medial gastrocnemius (MG) at 0% MVC (rest) and 100% maximum voluntary contraction (MVC). We manually digitized the cross points (Cross‐Pt) of the MG fascicle and deep aponeurosis (Apo) on each frame during ramp trials. The dark stripe is the shadow of the echo‐absorptive marker (Marker) attached to the skin. (b) An example of measured Cross‐Pt displacement (Raw), estimated the displacement due to ankle rotation (Due to Rotation) and corrected tendon elongation (Corrected). (c) An example of force‐tendon elongation curve. Tendon stiffness was calculated as the slope (red) of force and tendon elongation above 50% of MVC.

An examiner manually traced the AT in the transverse ultrasound images using open‐source image analysis software (ImageJ, NIH, Bethesda, USA). The same examiner manually digitized the cross point of the fascicle and deep aponeurosis using digitizing software (Frame Dias V, DKH, Tokyo, Japan) on each image in the ramp (Figure 1a) and passive trials.

MATLAB (version R2021b; The MathWorks, Inc., Massachusetts, USA) was used for further analysis. The digitized cross‐point coordinates, torque signals, and reflective marker coordinates were smoothed using a fourth‐order zero‐lag Butterworth low‐pass digital filter with a cutoff frequency of 8 Hz based on a residual analysis using digitized cross‐point coordinates.

The tendon force (F) was estimated using the ankle torque (τ) measured by the dynamometer and AT moment arm at 90° of the ankle joint (r) with the following equation:

$$F=\tau r^{-1}$$

At the moment, the arm was estimated from the shank length of each participant and using the scaling equation based on the study by Grieve et al. (1978), as previously described (Kubo et al., 2012).

The tendon elongation was measured as the displacement of the cross‐point coordinates. It is difficult to completely avoid ankle joint rotation during ankle MVC trials (Magnusson et al., 2001), which may lead to an overestimation of the measured tendon elongation. To avoid the effect of ankle rotation on tendon elongation, we corrected the tendon elongation using the motion‐capture‐based ankle joint angle profiles during the ramp trials and the movement of the fascicle–aponeurosis cross point during the passive trial (Figure 1b). The ankle joint angle was calculated as the angle between the lateral malleolus and the fifth metatarsal and lateral malleolus–fibula apex vectors. The tendon stiffness was calculated as the slope of the force and corrected tendon elongation above 50% of the maximum force of each ramp trial (Kubo et al., 2017) by using the least squares method (Figure 1c).

### 2.4 Statistical analysis

The means of the mechanical property data from the two ramp trials and CSA data from the three measurements were used as the representative values for each participant. To check the reliability of the AT CSA, AT stiffness, and MVC torque, we used intraclass correlation coefficients (ICC) with the following classifications: good (0.75–0.90) and excellent (>0.90) (Koo & Li, 2016). To quantify the magnitude of difference between groups, we calculated the high jumpers‐to‐controls ratio for AT stiffness and MVC torque. This ratio provides a standardized way to compare the relative differences across different variables and facilitates comparison with previous studies that have examined athletes versus nonathletes.

The normal distribution of each variable was confirmed using the Shapiro–Wilk test. Two‐tailed, independent *t*‐tests were used to determine the differences between the high jumpers and controls. The effect size of each *t*‐test was determined using Cohen's *d* for independent samples with the following classifications: very small (≤0.19), small (0.20–0.49), medium (0.50–0.79), large (0.80–1.19), very large (1.20–1.99), and huge (≥2.00) (Sawilowsky, 2009). As we could not match the body height (and thereby the shank length) between high jumpers and controls, we initially considered using analysis of covariance (ANCOVA) to account for its potential confounding effect on MTU mechanical properties. Prior to conducting ANCOVA, we examined the relationships between shank length and AT stiffness, as well as absolute and normalized MVC torque using Pearson's correlation coefficient.

To investigate the relationship between the high jump personal best record (PB) and MTU properties, we calculated Pearson's correlation coefficients. The level of significance was set at *α* = 0.05.

## 3 RESULTS

We confirmed the excellent reliability of the AT CSA (ICC = 0.92), MVC torque (ICC = 0.99), and AT stiffness (ICC = 0.94). Body height, as well as shank and AT lengths, was significantly greater in high jumpers than in controls (Table 1). No significant differences were observed in normalized AT length, body mass, and AT CSA between the groups (Table 1).

**TABLE 1:** Morphological characteristics of high jumpers and controls.

	High jumpers	Controls	*p*	*d*
	*n* = 10	*n* = 14		
Body height [m]	1.810 ± 0.049	1.706 ± 0.062	<0.001	1.82
Body mass [kg]	65.10 ± 6.45	64.60 ± 8.05	0.868	0.07
Shank length [mm]	417 ± 13	388 ± 18	<0.001	1.86
AT length [mm]	198 ± 21	179 ± 17	0.026	1.05
Normalized AT length [%]	47.4 ± 4.2	46.1 ± 4.8	0.510	0.27
AT CSA [mm^2^]	67.5 ± 10.9	64.1 ± 9.9	0.441	0.33

*Note*: Mean ± SD, *p* value of *t*‐test, and Cohen's *d*. Normalized AT length is the ratio of Achilles tendon length to shank length.

**FIGURE 2:**
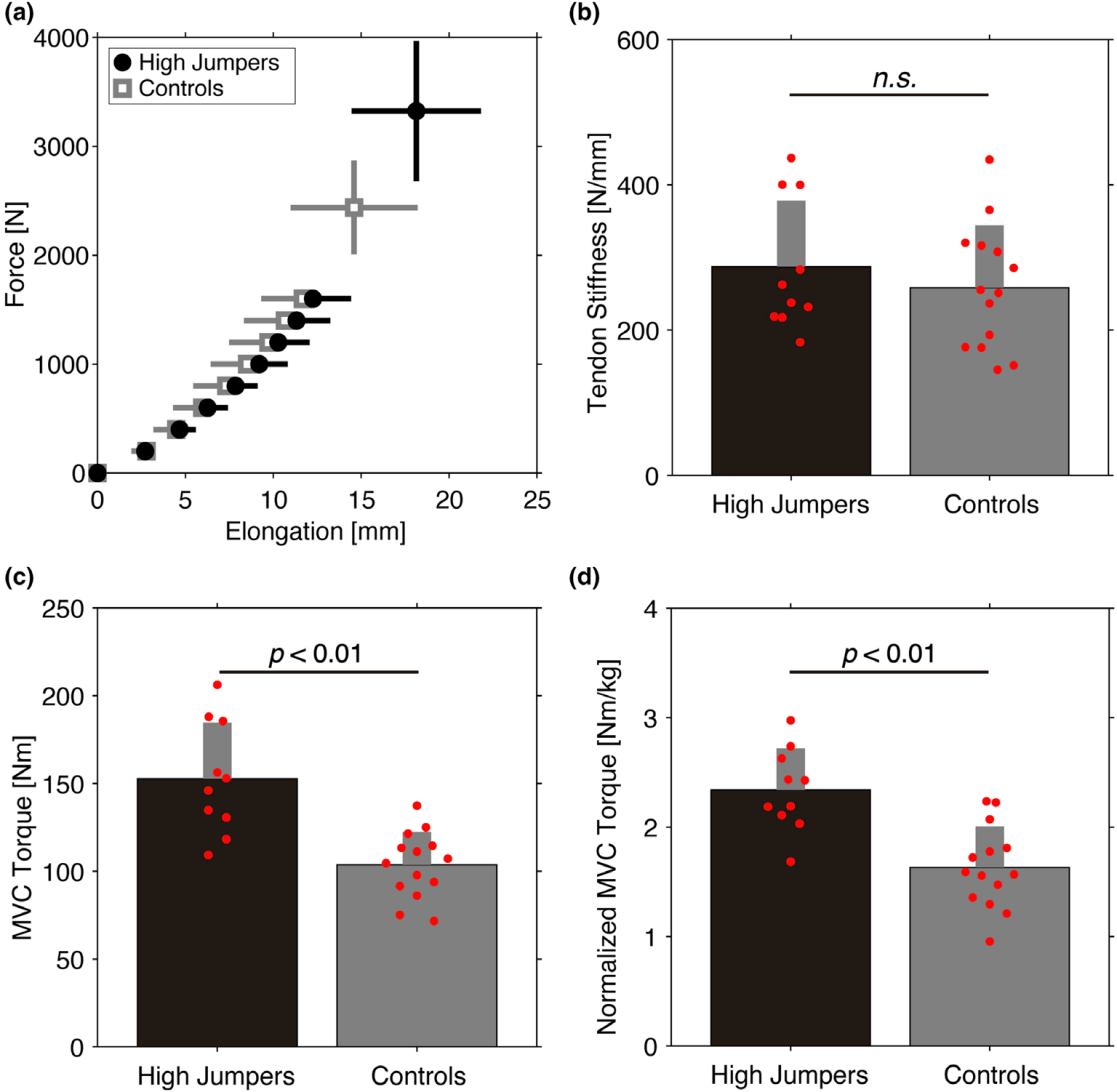
Mechanical characteristics of the high jumpers (black) and controls (gray). Mean, SD and individual data (red). (a) Elongation at every 200 N and maximum force during ramp trials. (b) Achilles tendon stiffness. (c) Maximum ankle plantar flexion torque. (d) Maximum ankle plantar flexion torque normalized by body mass. n.s., not significant.

The force‐elongation curves in the high jumpers and controls overlapped visually (Figure 2a). AT stiffness was not significantly different between the high jumpers (287.3 ± 90.9 N/mm) and controls (258.4 ± 85.6 N/mm) (*p* = 0.441, *d* = 0.33, high jumper‐to‐control ratio: +11.1%) (Figure 2b). The absolute and normalized MVC torques were significantly greater in the high jumpers (152.8 ± 31.8 Nm and 2.34 ± 0.38 Nm/kg) than in the controls (103.6 ± 18.9 Nm and 1.63 ± 0.38 Nm/kg) (*p* < 0.001 and <0.001, *d* = 1.97 and 1.88); the high jumper‐to‐control ratio for the absolute and normalized MVC torques was +47.5% and + 43.6%, respectively (Figure 2c,d). Shank length was not a covariate for either AT stiffness, absolute MVC torque, and normalized MVC torque (*r* < 0.58, *p* ≥ 0.079); thus, ANCOVA was not conducted.

**FIGURE 3:**
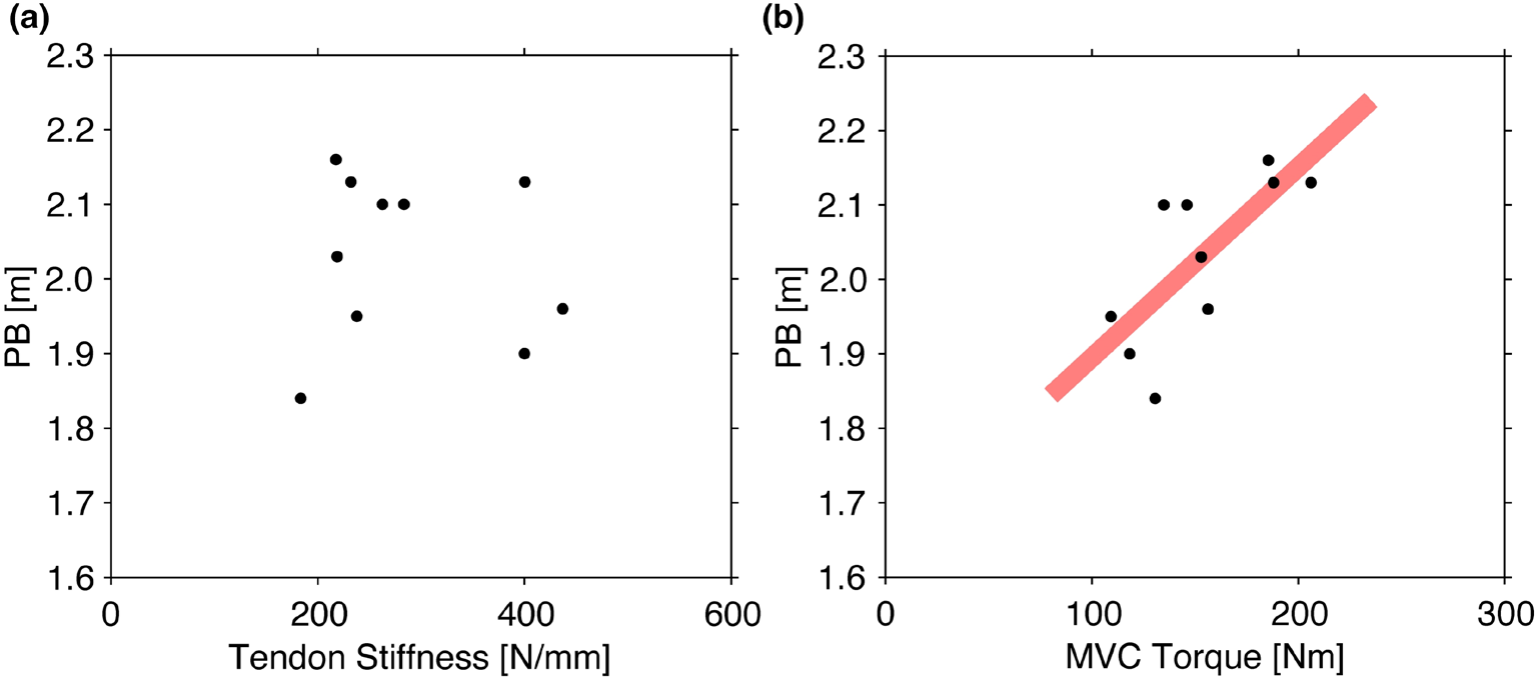
The relationship of high jump performance and MTU mechanical characteristics in high jumpers. (a) High jump personal best record and Achilles tendon stiffness. (b) High jump personal best record and maximum ankle plantar flexion torque.

AT stiffness was not significantly correlated with PB (*r* = −0.074, *p* = 0.839) (Figure 3a), whereas both absolute and normalized MVC torques were positively correlated with PB (*r* = 0.733 and 0.778, *p* = 0.016 and 0.008) (Figure 3b).

## 4 DISCUSSION

We found that the high jumpers exerted a greater ankle plantar flexion torque but did not have a stiffer AT than the controls, supporting our hypothesis. These findings differ from the previous comparison of sprinters and controls (Arampatzis et al., 2007) as well as most resistance training interventions (Wiesinger et al., 2015), which have shown athletes or training results having a stiffer AT. The AT stiffness of both the high jumpers and controls in the present study was similar to that of untrained populations in previous studies [180–280 N/mm (Arampatzis et al., 2010; Kubo et al., 2012)] using similar measurement methods. The standard deviation of AT stiffness was similar between the groups. The correlation between high jump PB and AT stiffness was not significant. These results suggest that high jumpers who participated in this study were not selectively filtered based on AT stiffness. Taken together, these results suggest that jumpers can acquire stronger muscles without significant changes in tendon stiffness. Regarding high jumpers as an example of a population of trained humans, our results imply that neuromuscular adaptations leading to enhanced force production can occur independently from mechanical adaptations in the tendon tissue, even in the long term.

In addition to AT stiffness, the intergroup differences in AT CSA and normalized AT length were not significant. The results suggest that neither morphological nor material adaptation has occurred in the AT of the high jumpers. The high jumpers in the present study had been training regularly for an average of 7.7 years and at least 4 years, training volume was 12.8 ± 2.7 h/week [range: 8–17 h/week], which would be suitable for examining long‐term tendon adaptation. Some short‐term interventional studies reported that plyometric training did not increase tendon stiffness (Houghton et al., 2013; Kubo et al., 2017); however, considering the slow turnover of tendon tissue (Heinemeier et al., 2013), the possibility remained that the non‐stiffening of the tendon was only temporary. However, our results reject this possibility and imply that muscles can be strengthened without stiffening tendons, even in long‐term adaptation.

The high jumpers exerted a + 47.5% greater MVC torque than controls with no significant difference in tendon stiffness. A greater rate of increase in muscle force than that in tendon stiffness indicates higher relative tendon compliance [defined as the maximum muscle force per tendon stiffness (Lichtwark & Barclay, 2010)]. In conditions of MTU rapid shortening, higher relative tendon compliance can facilitate muscle force exertion (Bohm et al., 2021; Fukashiro et al., 2006), thereby enhancing the MTU power exertion (Lichtwark & Barclay, 2010). Thus, improving muscle strength without tendon stiffness could be advantageous for human motor tasks with exerting great power. Meanwhile, considering the larger tendon strain as a factor in tendon damage (Wren et al., 2003), increased relative compliance may increase the risk of tendon injury. The upper limit of tendon strain is approximately 10% (Wang, 2006); exceeding this limit may cause tendon rupture. Therefore, greater relative tendon compliance in high jumpers may increase the risk of tendinopathy or tendon rupture. The findings provide a novel rationale for further studies on tendon injuries.

MVC torque was significantly correlated with high jump PB (*r* = 0.733). This suggests that the ability to exert great muscle force is essential for jumping high. From a mechanical perspective, the ability to exert great ankle plantar flexion torque could be an advantage for high jump performance, as it has the potential to perform large joint work during take‐off. A previous motion analysis (Sado et al., 2020) revealed that the mechanical energy generated by ankle work during the takeoff phase is translated into jump height in the high jump. We further add that the torque exertion ability of the ankle plantar flexors is an important explainer for the interindividual variability in jumping performance. Meanwhile, tendon stiffness was not correlated with high jump PB (*r* = −0.074). Furthermore, there was no significant difference between high jumpers and untrained populations in AT stiffness. These results suggest that humans do not need to stiffen the AT or increase the AT extensibility from our natural stiffness to jump high. This means that humans inherently possess suitable AT mechanical properties for jumping, even without training. The human musculoskeletal system was primarily thought to be suited for sub‐maximum locomotion such as long‐distance running and walking (Bramble & Lieberman, 2004). Bohm et al. (2021) showed that tendon compliance leads to optimal muscle conditions with high force‐length potential during running, suggesting that human MTU is designed to minimize the metabolic cost of running. Our findings further suggest that humans have acquired tendon mechanical properties that are capable of functioning well not only in sub‐maximum but also in maximal motor tasks such as jumping.

Arampatzis et al. (2007) reported that the sprinter‐to‐control ratio for the MVC torque (+40.7%) was less than that for the tendon stiffness (+69.9%). Thus, although jumping and sprinting are both typical power‐demanding motor tasks in the SSC, the relative tendon compliance is high in high jumpers and low in sprinters. Interestingly, the different characteristics of relative tendon compliance are suitable for each temporal demand (i.e., ground contact time); sprinters with stiffer tendons require a short ground contact time, whereas high jumpers with non‐stiffer tendons do not. The effect of tendon stiffness on ground contact time has been partially reported (Abdelsattar et al., 2018), yet further study needs to be done to clarify the optimal stiffness for the temporal demands of each motor task. The difference in regular training programs between jumpers and sprinters might be informative for controlling relative tendon compliance, which should be investigated in future studies. Karamanidis and Epro (2020) reported that both increasing muscle strength and stiffening tendon occurred in jumpers [sprinter‐to‐control ratio; MVC torque (∼+20%) Tendon stiffness (∼+15%)], which was different from our findings. Karamanidis and Epro (2020) mixed jumpers with all jumping events (high jumpers *n* = 22, other events jumpers *n* = 45) as one group, while we examined only high jumpers. Long jumpers are required to run fast in the approach phase (Hay et al., 1986) and to complete the take‐off motion with a shorter duration than high jumpers (Wilson et al., 2011); thus, their training may be similar to that of sprinters. To investigate the differences in the long‐term adaptation of muscles and tendons in more detail, future studies might need to record and compare the loads imposed by training more precisely.

We have some methodological limitations. First, we used a cross‐sectional experimental design, and the potential contribution of genetics or natural selection to our data cannot be explicitly ruled out. Second, measured MVC torque is influenced by not only muscle properties but also neural factors. Thus, the greater MVC torque in high jumpers may include not only muscular morphological and mechanical adaptations but also neural adaptations. Third, there were significant differences in body height and shank length between the groups because we recruited the participants based on their body mass. As tendon stiffness is determined from the length and force variables, the differences in shank length (high jumper‐to‐control ratio: +7.7%) may influence the interpretation of AT stiffness. However, the tendon stiffness ratio (high jumper‐to‐control ratio: +11.1%) was much smaller than the MVC torque ratio (high jumper‐to‐control ratio: +47.5%). Furthermore, shank length was not a covariate in either AT stiffness or absolute MVC torque and normalized MVC torque. Thus, the difference in shank length between the groups does not critically affect our findings. Fourth, we measured the AT CSA at the lateral malleolus height following previous studies (Kubo et al., 2020); we could not conclude on the morphological changes across the entire AT, as adaptation in AT thickening is inhomogeneous. The validity of measuring CSA using B‐mode ultrasound is also controversial (Kruse et al., 2017). These limitations must be considered when interpreting our data.

In conclusion, our findings suggest that male high jumpers develop greater ankle plantar flexion torque without a corresponding increase in Achilles tendon stiffness. This implies that, at least in the context of high jump training, muscle strength can be enhanced independently of tendon stiffening, even with long‐term training spanning several years. Furthermore, improving muscle strength can be a key factor for jump performance, while modifying tendon stiffness may not be necessary. Humans may inherently possess adequate tendon properties for jumping, even without specific training.

## FUNDING INFORMATION

Japan Society for the Promotion of Science (JSPS): Toshihide Fujimori, 24KJ0479; Japan Society for the Promotion of Science (JSPS): Natsuki Sado, 21K17592. This study was supported by Japan Society for the Promotion of Science (JSPS) KAKENHI (24KJ0479 and 21K17592).

## CONFLICT OF INTEREST STATEMENT

The authors declare there are no competing interests.

## ETHICS STATEMENT

All experimental procedures performed in this study were approved by the Human Subjects Committee of the University of Tsukuba, Japan (reference number: 021‐95).

## Data Availability

The data that support the findings of this study are available from the corresponding author upon request.
